# Risk of airway fire with the use of KTP laser and high flow humidified oxygen delivery in a laryngeal surgery model

**DOI:** 10.1038/s41598-021-04636-3

**Published:** 2022-01-11

**Authors:** Lucy Huang, Adam Badenoch, Marthinus Vermeulen, Shahid Ullah, Charmaine Woods, Theodore Athanasiadis, Eng Hooi Ooi

**Affiliations:** 1 Department of Otolaryngology Head and Neck Surgery, Flinders Medical Centre, Bedford Park, SA Australia; 2 College of Medicine and Public Health, Flinders University, Bedford Park, Australia; 3 Department of Anaesthesia and Pain Medicine, Flinders Medical Centre, Bedford Park, Australia

**Keywords:** Translational research, Risk factors

## Abstract

Airway surgery presents a unique environment for operating room fire to occur. This study aims to explore the factors of combustion when using KTP laser with high flow oxygen in an ex-vivo model. The variables tested were varying tissue type, tissue condition, oxygen concentration, laser setting, and smoke evacuation in a stainless-steel model. Outcome measures were time of lasing to the first spark and/or flame. A multivariate Cox proportional hazard model was used to determine the risk of spark and flame across the different risk factors. For every 10% increase in oxygen concentration above 60% the risk of flame increased by a factor of 2.3. Continuous laser setting at 2.6 W increased the risk by a factor of 72.8. The risk of lasing adipose tissue is 7.3 times higher than that of muscle. Charred tissue increases the risk of flame by a factor of 92.8. Flame occurred without a preceding spark 93.6% of the time. Using KTP laser in the pulsed mode with low wattages, minimising lasing time, reducing the oxygen concentration and avoiding lasing adipose or charred tissue produce a relatively low estimated risk of spark or flame.

## Introduction

Operating-room airway fires are serious and potentially fatal complications but fortunately rare in otolaryngologic surgery^1,2^. Airway fires have been described during adenotonsillectomy, tracheostomy and endoscopic airway surgery^3^. The triad of essential elements required for an airway fire are oxygen, fuel, and an ignition source^4^. Oxygen is in abundance in a shared airway setting where it may be delivered via a facemask, traditional nasal cannulae, supraglottic jet ventilation, endotracheal tube, or high-flow nasal oxygen. The ignition source is usually from the heat generated from electrocautery devices or lasers. Potential fuel sources include endotracheal tubes (ETT), gauze, surgical drapes and the eschar from charred tissue^2,5^.

Transnasal humidified rapid insufflation ventilatory exchange (THRIVE) is a method of oxygenating patients in a shared airway setting by delivering oxygen at a high flow rate to the lungs via nasal cannulae without an endotracheal tube. It allows for an unobstructed surgical field^6^ and eliminates the endotracheal tube as a potential fuel for ignition^7^. Nasal high-flow oxygen used for THRIVE usually delivers 100% oxygen, however devices incorporating oxygen-air blenders are now available to alter the oxygen concentration delivered. Reports of fires during the use of nasal high flow oxygen in conjunction with electrocautery and laser are also emerging^8–11^.

The use of lasers in laryngeal surgery has increased over recent years, delivering targeted resection with improved haemostasis^12^. The traditionally used carbon dioxide (CO_2_) laser has a wavelength of 10,600 nm, which is strongly absorbed by water and tissue and is associated with most airway fire reports to date^4,13,14^. By contrast, Potassium-titanyl-phosphate (KTP) laser is a solid-state laser with a wavelength of 532 nm, resulting in specific absorption of energy by oxyhaemoglobin in red blood cells, producing photoangiolysis of blood vessels at lower tissue temperature with less vaporisation^12,15^. These factors may theoretically reduce the risk of an airway fire when used in upper airway surgery, compared with the CO_2_ laser. Studies examining the factors that contribute to the risk of airway fires in clinical practice have largely been conducted using the CO_2_ laser^4,16–18^. The study by Roy et al. used a mannequin intubated with a laser safe ETT, demonstrating an immediate sustained fire when the CO_2_ laser struck and perforated the ETT cuff. This occurred with oxygen concentration as low as 40% indicating that traditional methods of airway management with an ETT for upper airway laser use are not risk free^3^. Dhar et al. studied CO_2_ laser in combination with subglottic jet ventilation in a porcine model, identifying oxygen concentration, laser power and dry fuel source as independent risk factors for combustion^16^. A subsequent study by Stuermer et al. confirmed laser power and oxygen concentration as risk factors and identified tissue type (adipose, cartilage, muscle), tissue quality (fresh, charred) and the use of smoke evacuation as additional independent risk factors for combustion in an ex-vivo plexiglass model using CO_2_ laser^4^. These two studies were conducted using non-humidified oxygen sources at flow rates significantly lower (jet ventilation at 2 bar and low flow rate at 10L/minute) than those generated when using THRIVE (70L/min)^4,16^. It is possible that 100% humidification of the gas mixture and the high flow rate provided by THRIVE reduces the risk of combustion^18^.

There has been no study investigating the risk of KTP laser causing an airway fire despite its common use in laryngeal surgery. The aim of this study is to examine the factors associated with combustion when using KTP laser in combination with humidified high-flow nasal oxygen. We developed an ex-vivo model which is designed to simulate laryngeal surgery to facilitate risk prediction of spark and flame under clinically relevant conditions.

## Methods

Human ethics was not required for this study as all experiments were performed using animal tissue (porcine muscle and adipose tissue) purchased from the local butcher. This study is listed on the Animal Register at Flinders University.

**Figure 1:**
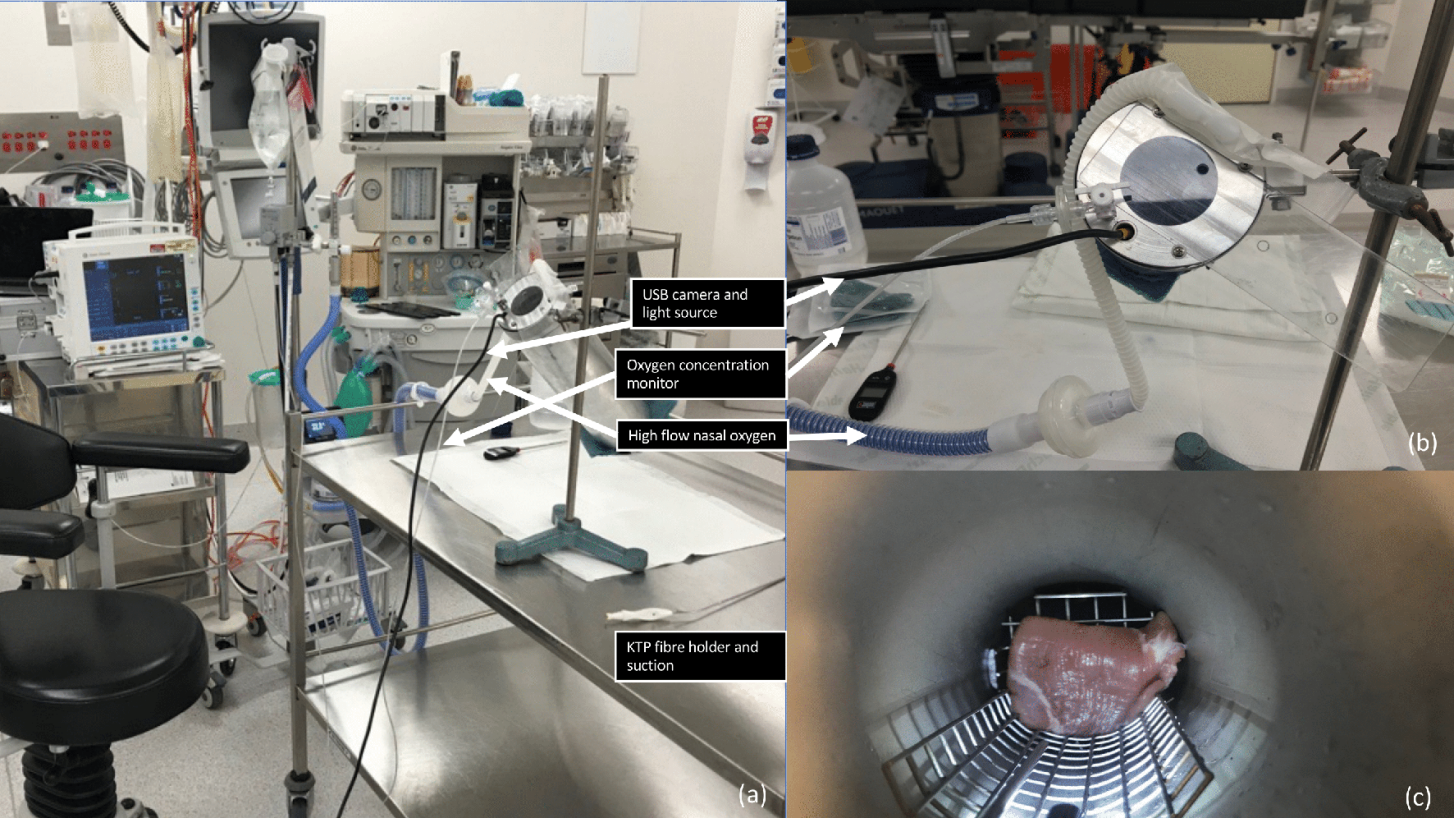
Experimental setup (**a**) Experimental setup with the stainless steel laryngeal model in an operating theatre environment with Optiflow™; (**b**) The inlet of the laryngeal model with a plastic covering, Optiflow™ nasal cannula, oxygen sensor probe and USB camera; (**c**) The internal view of the model with a stainless-steel platform holding the porcine specimen.

The ex-vivo experiment took place in the operating theatre using standard anaesthetic and operating equipment. A model was designed by the Biomedical Engineering Department to simulate the upper airway, consisting of a cylindrical stainless-steel chamber (outer diameter: 10 cm, inner diameter: 5 cm, length: 32 cm; Fig. 1a,b). Positioned superiorly, are openings for the Optiflow™ nasal canula, KTP laser fibre holder, suction, endoscopic camera, and an oxygen sensor to verify precise tissue oxygen concentration at the point of lasing. A plastic covering was used to reduce oxygen loss and maximise oxygen concentration within the chamber. Inferiorly, positioned 25 cm from the opening is a metal platform that holds the tissue being lasered (Fig. 1c). The Aura XP Laser Therapy System™ (Boston Scientific) was used with EndoStat 0.4 mm laser fibres, using a non-contact firing technique. Optiflow™ (Fisher & Paykel), incorporating an oxygen-air blender, was used to provide flow rates of 70L/minute (min), simulating THRIVE with the ability to alter the oxygen concentration.

**Figure 2:**
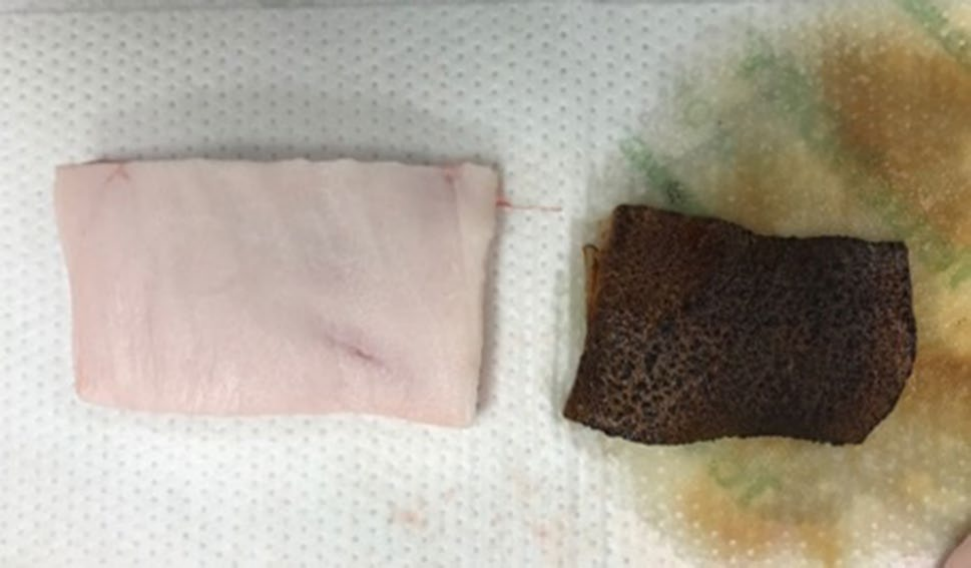
An example of uncharred and charred adipose tissue. Approximate size measuring 8 cm × 4 cm for the uncharred adipose tissue, 6 cm × 3.5 cm for the charred adipose tissue.

Porcine adipose and muscle tissue were used, as they represented the most and least flammable tissue type in the study performed by Stuermer et al.^4^. The KTP laser settings were chosen based on clinical relevance (2.6 W continuous, 5 W continuous, 26 W pulsed and 35 W pulsed settings; pulsed setting set at 15 ms pulse width, 2 pulse per second)^19^. Lasing was performed on both charred and uncharred, adipose and muscle tissue with variable oxygen concentrations (30%, 40%, 50%, 60%, 70%, 80%, 90%), with and without smoke evacuation. Each piece of tissue was placed in a plastic bag and warmed using a water bath to 30 °C. Charring of tissue was conducted using a kitchen blow torch to enable even charring on tissue surface (Fig. 2). 100% humidification and gas flow rate at 70L/min were used in every instance. Each combination of variables was repeated at least five times to ensure reproducibility. If a flame occurred, oxygen was immediately reduced to 21% using the oxygen-air blender while maintaining 70L/min flow rates, which rapidly extinguished the flame. Example videos have been provided in Supplementary video 1 and 2.

### Outcome measures

The outcomes were the occurrence of a spark and/or flame. Flame is considered the most important patient-centred outcome; however, sparks are often used clinically as a surrogate marker to indicate increased risk of flame if lasing continues, therefore spark and flame were modelled separately from the same data set. Time was measured from the start of lasing to the time to first spark/flame, or until 60 s. A 60 s cut off time was used, as lasing beyond 60 s is not clinically relevant.

### Statistical methods

A multivariate Cox proportional hazard model was used to examine the risk of spark and flame across oxygen concentration, laser setting, tissue type, charring and smoke evacuation. Time to event was measured from the time of lasing onset to the time of spark/flame. Experiments were censored at 60 s.

$$ h\left( {t,z,x} \right) = Zh_{0} \left( t \right)exp\left( {\beta^{T} X} \right) $$

Analytically, the hazards rate was defined from the Cox proportional hazard model where $h_{0} \left( t \right)$ is the baseline hazard function; $\beta$ is the vector of regression coefficients; X is the vector of observed covariates (oxygen concentration, laser setting, tissue type, charring and smoke evacuation) and Z is the frailty variable. The parameters were estimated by the maximum likelihood method. As each combination of experimental conditions was repeated 5 times, the models captured the mixed effect using shared frailty—the same random effect is shared by all repeated events within the same experimental group. The frailties are assumed to be gamma-distributed latent random effects that affect the hazard multiplicatively. The variables included in the final multivariate model were oxygen concentration, laser setting, tissue type and charring. The inclusion criterion was based on clinical importance, and statistical significance at *p* ≤ 0.20 from a univariate model. Variables tested experimentally and included in the univariate analyses were all previously identified as independent risk factors in previous studies using different lasers^4,16,18^. Smoke evacuation was subsequently excluded from the multivariable model based on the non-significance in the univariate model. Additionally, re-inclusion of smoke evacuation into the final model did not result in a statistically significant association or change in the model coefficients. Proportional hazard assumption was tested by log–log plot of survival and Schoenfeld residuals; it was found to be valid. Linear splines were employed to account for non-linearity of association between oxygen concentration and outcome hazard. The spline cut-off at 60% oxygen was selected based on it being an inflection point in the observed data and a judgement that this cut-off was clinically relevant to the risk of hypoxia when THRIVE is used clinically. Cumulative hazard curves were evaluated using the standard Nelson-Aalen cumulative hazard functions.

The clinically relevant probabilities of spark or flame were established using the final multivariable Cox proportion hazard models by restricting the predictive model to 5 s of lasing (or time to event if < 5 s) to facilitate a pragmatic and clinically relevant risk assessment. Probabilities were generated based on calculation of coefficients from the regression model and estimates of 5-s risk of spark or flame. The following equation was built from the coefficients (log HRs) of multivariate cox proportional hazards model for flame. In particular, the coefficient was multiplied by the value of the variable and then summed for each variable to get the scores (L). The baseline survival function was then exponentiated by the score and then subtracted from 1 to calculate the 5-s risk of flame. A similar approach can apply for calculating the risk of spark.

$$ \begin{aligned} L & = 0.24 \times oxygen\;concentration < 60\% - 0.09 \times oxygen\;concentration \\ & \ge 60\% + 4.40 \times continuous\;2.6\;{\text{W}}\;laser\;mode \\ & \quad + \;3.52 \times continous\;5\;{\text{W}}\;laser\;mode + 1.72 \times Pulsed\;35\;{\text{W}}\;laser\;mode \\ & \quad + \;2.79 \times adipose\;tissue\;type. \\ \end{aligned} $$

Finally, the adjusted predictions were displayed after using a restricted cubic spline to account for non-linear relationships between oxygen concentration and the risk of spark or flame. Model diagnostics and goodness of fit were evaluated by Harrell's C concordance statistic. The two-sided test was performed for all analysis, 95% confidence intervals were reported and the level of significance was set at *p* < 0.05.

## Results

The ex-vivo testing resulted in 2182 firing episodes across a total of 224 combinations of variables. There were no missing data.

Flames were preceded by sparks in 6.4% of instances and occurred without sparks 93.6% of the time. In those instances where sparks preceded flames the median (25th–75th percentiles) time difference between spark and flame was 19 (4–44) seconds.

Notably, no sparks or flames were observed when laser firing was limited to 5 s at fresh tissue irrespective of oxygen concentration, laser setting or tissue type.

**Figure 3:**
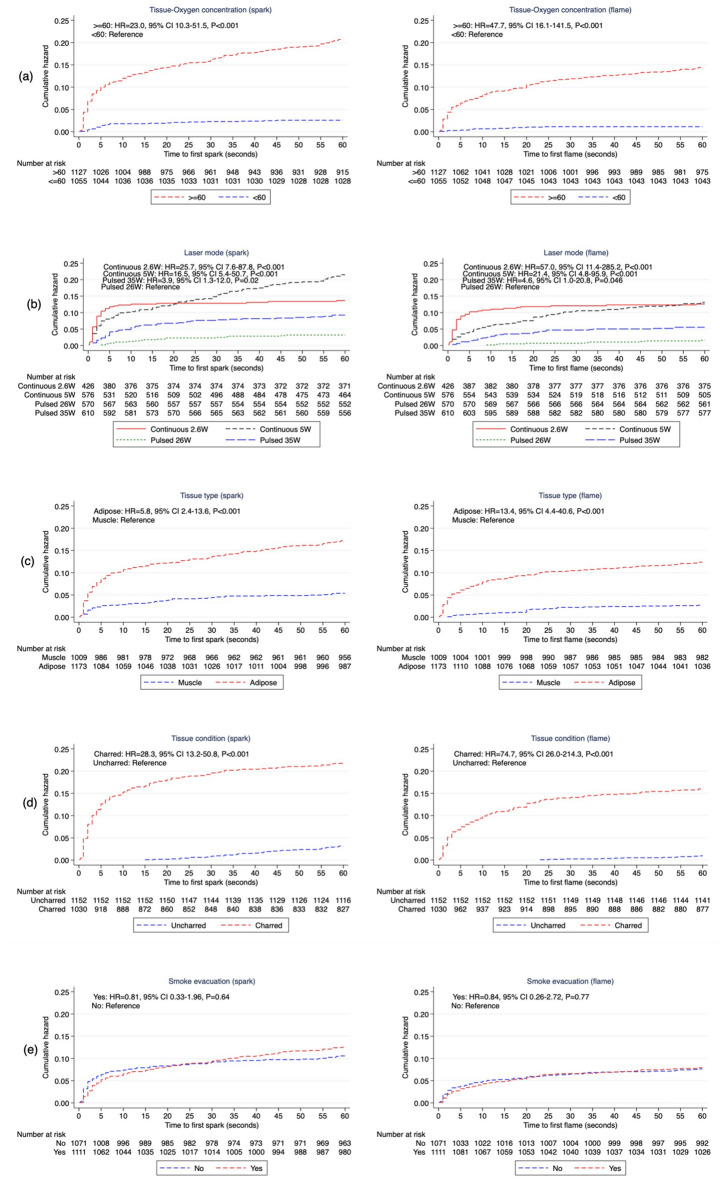
Nelson-Aalen cumulative hazard function of spark and flame by (**a**) oxygen concentration (**b**) laser mode (**c**) tissue type (**d**) tissue condition (**e**) smoke evacuation based on ex-vivo experimental data.

**Figure 4:**
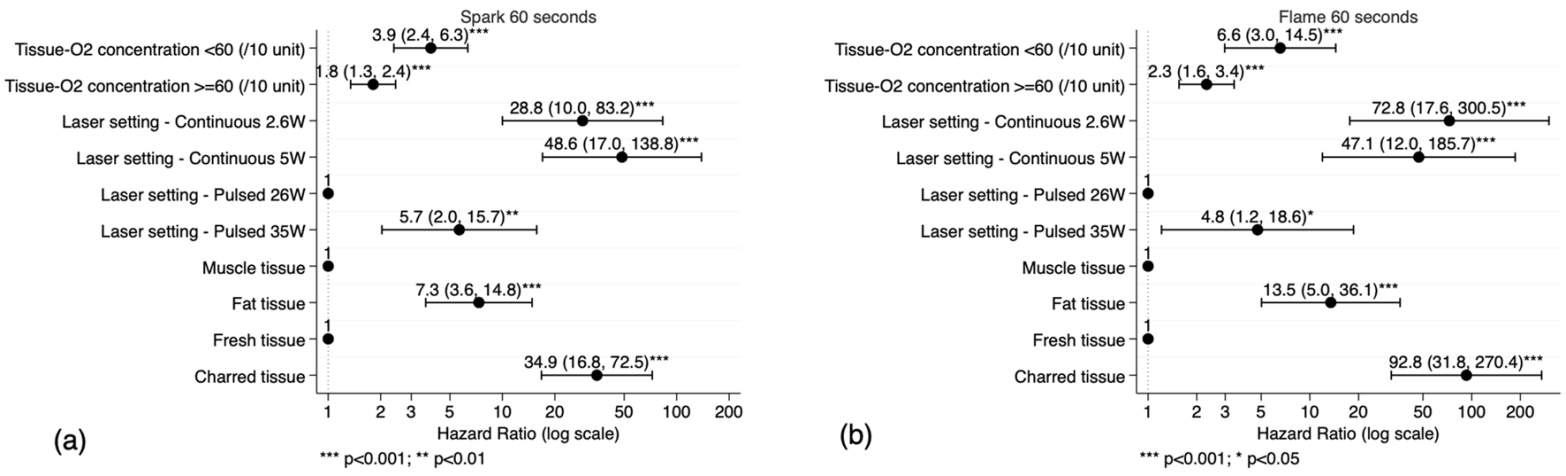
Coefficient plot from multivariate Cox proportional hazard models of (**a**) spark and (**b**) flame censored after 60 s from ex-vivo experimental data.

Oxygen concentration, laser mode, tissue type, and tissue quality were all independent risk factors for spark and flame (Fig. 3a–d). Smoke evacuation was not a significant predictor of spark nor flame in either the univariate or multivariate analyses and was therefore excluded from the final multivariable model (Fig. 3e). Results of the final multivariable model for spark and flame are presented in (Fig. 4). The Harrell's C concordance statistic for the multivariable model was 0.91 for spark and 0.94 for flame.

When compared with uncharred tissue, the risk (95% CI) of spark when lasing at charred tissue was increased by 34.9 (16.8–72.5) (*p* < 0.001) and the risk of flame increased by 92.8 (31.8–270.4) (*p* < 0.001) (Fig. 4b).

For oxygen concentration within the range 25–59%, the risk (95% CI) of spark increased by 3.9 (2.4–6.3) (*p* < 0.001) and flame increased by 6.6 (3.0–14.5) (*p* < 0.001) for every 10% increase in oxygen concentration. For oxygen concentration within the range 60–90%, the risk of spark increased by 1.8 (1.3–2.4) (*p* < 0.001) and flame increased by 2.3 (1.6–3.4) (*p* < 0.001) for every 10% increase in oxygen concentration.

Compared with pulsed 26 W (lowest power setting), pulsed 35 W increased the risk (95% CI) of spark by 5.7 (2.0–15.7) (*p* < 0.01) and the risk of flame by 4.8 (1.2–18.6) (*p* < 0.05). The risk (95% CI) of spark at continuous 2.6 W and 5 W are increased by 28.8 (10.0–83.2) (*p* < 0.001) and 48.6 (17.0–138.8) (*p* < 0.001) respectively. The risk (95% CI) of flame at continuous 2.6 W and 5 W are increased by 72.8 (17.6–300.5) (*p* < 0.001) and 47.1 (12.0–185.7) (*p* < 0.001) respectively. There was no statistically significant difference between the risk of spark or flame associated with 2.6 W and 5 W continuous settings when compared with each other.

Compared with muscle, the risk (95% CI) of spark when lasing at adipose tissue is 7.3 (3.6–14.8) (*p* < 0.001) times higher and the risk of flame is 13.5 (5.0–36.1) (*p* < 0.001) times higher.

### Clinical risk estimates

**Figure 5:**
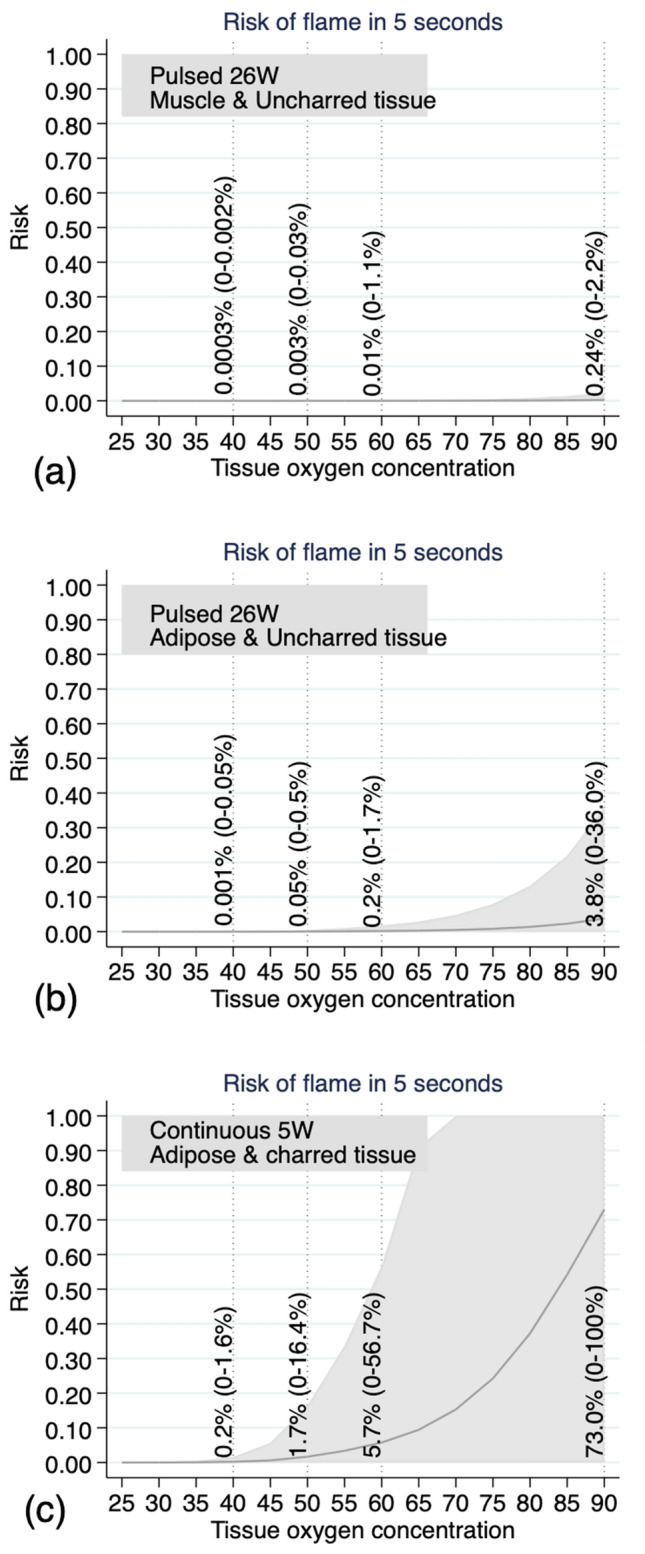
Estimated clinical risk of flame when lasing up to 5 s by oxygen concentration, laser mode, tissue type and charring based on experimental data. Percentages on the figures show the risks for oxygen concentration 40, 50, 60, and 90 respectively. (**a**) Laser setting: 26 W pulsed, tissue: uncharred muscle; (**b**) Laser setting: 26 W pulsed, tissue: uncharred adipose tissue; (**c**) Laser setting: 5 W continuous, tissue: charred adipose tissue.

When modelling specific, clinically relevant scenarios, the risk (95%CI) of flame estimated when using 26 W pulsed settings with 40% oxygen was 0.0003% (0 to 0.002) (Fig. 5a) on uncharred muscle and 0.001% (0 to 0.05) on uncharred adipose tissue (Fig. 5b). As a comparison, the most flammable combination (5 W continuous setting on charred adipose tissue) demonstrated the likelihood of flame within 5 s is as high as 0.2% (0–1.6) with 40% oxygen and 5.7% (0–56.7) with 60% oxygen (Fig. 5c). The inflection point for increased risk of flame with increasing oxygen varies depending on the specific combination of variables (laser mode, tissue type & charred status) (Fig. 5).

Similar estimates were determined for the occurrence of spark (Supplementary Fig. S1). The least flammable combination (laser at 26 W pulsed setting, on uncharred muscle) (Supplementary Fig. S1a) resulted in an estimated risk (95%CI) ranging from 0.01% (0–0.04) at 40% oxygen to 0.09% (0–0.5) at 60% oxygen. The most flammable combination (laser at 5 W continuous setting, on charred adipose tissue) result in an estimated risk (95%CI) ranging from 1.8% (0–9.8) at 40% oxygen to 20.6% (0–100) at 60% oxygen.

## Discussion

This is the first study to evaluate the risk of fire when using KTP laser in a high flow oxygen setting. Theoretically fire risks are reduced when THRIVE is used with the removal of potential fuel sources, the humidification of gases within the surgical field and the provision of an effective mechanism to remove laser smog. Furthermore, KTP laser has theoretical advantages over CO_2_ laser in terms of reduced tissue heating dynamics^12^. However, this study demonstrates that these factors do not negate the possibility of an airway fire. Factors comprising of continuous laser mode, oxygen concentration greater than 60%, increased adiposity and the presence of char provide increased risk estimates of spark or flame, with many instances of flame occurring without a preceding spark. Native tissue that is high in adiposity or has been charred during the surgical process are highly combustible, and therefore a high level of vigilance is required of the surgeon to remove any charring when lasing with THRIVE.

Oxygen concentration is an important predictor of the risk of spark and flame. An important clinical question is whether the oxygen concentration required to prevent airway fires is compatible with adequate patient oxygenation. The current recommendation from the Joint Commission on Accreditation of Healthcare Organizations is to use an oxygen concentration of less than 30% when delivered in an open manner during facial surgery^20^. Despite this recommendation, Roy & Smith were able to ignite a non-sustained flame when their laser struck and perforated the cuff of a laser safe endotracheal tube, using 5 W CO_2_ laser with an oxygen concentration of 29% for an unspecified length of time^3^. Importantly, the wider pulse width of the KTP laser allows slower heating, providing a theoretical smaller risk of combustion compared to CO_2_ laser^12^. The current study demonstrates that clinically relevant combinations of oxygen concentration, laser setting and lasing duration result in reasonably low estimated risks; but these may still be considered too high in the clinical setting given the devastating consequences^21^ if airway fire is to occur. For example, an estimated risk of flame is 0.05% (5 fires per 10,000 lasing) with an upper limit of the 95% CI of 0.5% (5 fires per 1,000 lasing) at 50% oxygen with a 26 W pulsed setting on uncharred adipose tissue. This risk is reduced when oxygen concentration is reduced to 40% (risk of 0.001% with an upper limit of 95% CI of 0.005%) or if the tissue type is uncharred muscle (estimated risk of 0.003% with upper limit of 95% CI of 0.03%). The oxygen concentration delivered during KTP laser use should be titrated according to patient’s oxygen requirements with the potential for delivering higher oxygen concentration when using pulsed KTP settings of lower power. We did not observe any sparks or flames when lasing for less than 5 s on uncharred tissue irrespective of oxygen concentration, laser power or tissue type. This indicates that KTP laser and the absence of a fuel source, such as an ETT, reduces the risk of combustion. Furthermore, lasing for less than 5 s at a time is highly unlikely to result in combustion. The consequences of an airway fire are severe; therefore, surgeons and anaesthetists should work together with the aim to minimise this risk as much as possible while balancing the risk of hypoxia. An oxygen-air blender can rapidly alter oxygen concentration, providing low oxygen fractions during periods of lasing. Further in-vivo studies are needed to determine the oxygen concentration at the level of the larynx when using an oxygen-air blender with THRIVE.

Smoke evacuation using suction was identified as an important factor to reduce combustion of vaporised carbonised tissue (i.e. laser smog) when using CO_2_ laser^4^. However, smoke evacuation was not demonstrated as an important factor in the current study. This may reflect the different tissue absorption characteristics of KTP laser with reduced surface temperature, resulting in less tissue vaporisation compared to the CO_2_ laser^12^. Alternatively, the high flow rate of THRIVE at 70L/minute may also mean additional smoke evacuation via suction is redundant. In addition to removing laser smog, the high flow rate may also provide continuous positive pressure to reduce atelectasis and shunting of deoxygenated blood through the lungs when used in-vivo which may maintain oxygenation when using lower oxygen concentration. This is demonstrated in one previous study that an oxygen flow rate of 50L/min delivered via the nose is able to provide a positive airway pressure of 7 cm H_2_O^22^.

Spark is commonly used as a warning sign of flame; however, it is important to note that most flames occurred spontaneously without a preceding spark. When a spark did precede a flame, the time between spark and flame was highly variable, often less than 4 s. This suggests that sparks should not be used as a reliable indicator of imminent risk of sustained flame.

The continuous laser settings of 2.6 W and 5 W produced dangerously high clinical risk estimates of spark and flame. Despite 2.6 W appearing to be of higher risk than 5 W there was no statistical difference in the magnitude of these risk estimates with significant overlap of the 95% confidence interval. Regardless of the estimated clinical risk presented in this study, it would not be advisable to use either of these settings when using KTP laser in conjunction with THRIVE.

The flames that occurred in this study were all successfully extinguished by turning the oxygen concentration down to 21% using the oxygen-air blender except for one episode where embers caught a piece of dry gauze underneath the apparatus. This was rapidly extinguished with water. This serves as a reminder that reducing the concentration of oxygen along with adequate safety preparation prior to commencing laser airway surgery are important safety steps in the event of an airway fire. It was also noted that when flame was ignited on charred tissue, it rapidly spread to other sections of the tissue. The spread of the flame can be prevented by removing charred tissue from within the surgical field.

## Limitations

This model does not fully simulate a human respiratory system with gases entering the lungs and exiting along the same pathway (trachea), as most of the gas in this system escaped from the inferior end of the model. It also does not simulate the gas absorption that occurs in the human lungs. To replicate the gas flow in the respiratory system, the use of anaesthetised live animal models or a sophisticated computational fluid dynamic model with 3D printing^23^ will be expensive and likely be destroyed during the repetitive episodes of flame ignition. Therefore, ascertaining the concentration of oxygen intra-operatively at the level of the patients’ larynx with an oxygen measurement probe during upper airway procedures using THRIVE is an important next step to translate this data into meaningful clinical recommendations.

It would not be ethical nor practical to perform this study using live animals with the risk of airway fires and number of repeat experiments (> 2000 lasing) done to ensure reproducibility. Porcine tissue is frequently used as an analogue for human tissue in forensic sciences^24^ and were used instead of human larynges. This study chose the most and least flammable tissue types^4^, allowing us to present the range of risks associated with using KTP laser in this setting. The flammability characteristics of the surface mucous membrane during laryngeal surgery will be different to either adipose or muscle tissue, but will fall within the described range of risk prediction. Each porcine specimen may not have the exact same consistency of adiposity or muscle for each firing, but the chosen specimens were largely of the same tissue type macroscopically. KTP laser has an affinity for oxyhaemoglobin and as the tested tissue is non-living and has no circulating blood, this aspect could not be tested.

## Conclusion

There remains a high risk of combustion when using KTP laser in a humidified oxygen rich environment especially with high oxygen concentration, charring and adiposity. Extreme caution should be taken to reduce or eliminate these factors when using KTP laser in a shared airway setting. When combining high flow oxygen with KTP laser for upper airway surgery with standard clinical equipment in a real operating room environment the following combinations provided low estimated risk of spark or flame: KTP laser in the pulsed mode with low wattages, minimising lasing time, reducing the oxygen concentration and avoiding lasing adipose or charred tissue. Sparks should not be relied on as reliable indicators of imminent flame. As it is difficult to design a model to fully simulate gas flow in a human respiratory tract and allow for repetitive testing, it is important to conduct further studies by measuring laryngeal oxygen concentration in patients to translate this data into clinical practice.

## Supplementary Information

Supplementary Figure S1.

Supplementary Legends.

Supplementary Video 1.

Supplementary Video 2.

## Data Availability

The datasets used and analysed during the current study are made available via https://doi.org/10.6084/m9.figshare.17126285 once the study is published.
