# Supplementary figures and images for: Risk of airway fire with the use of KTP laser and high flow humidified oxygen delivery in a laryngeal surgery model
Source: Sci Rep. 2022 Jan 11;12:543. doi: 10.1038/s41598-021-04636-3 (PMC8752812; doi:10.1038/s41598-021-04636-3)

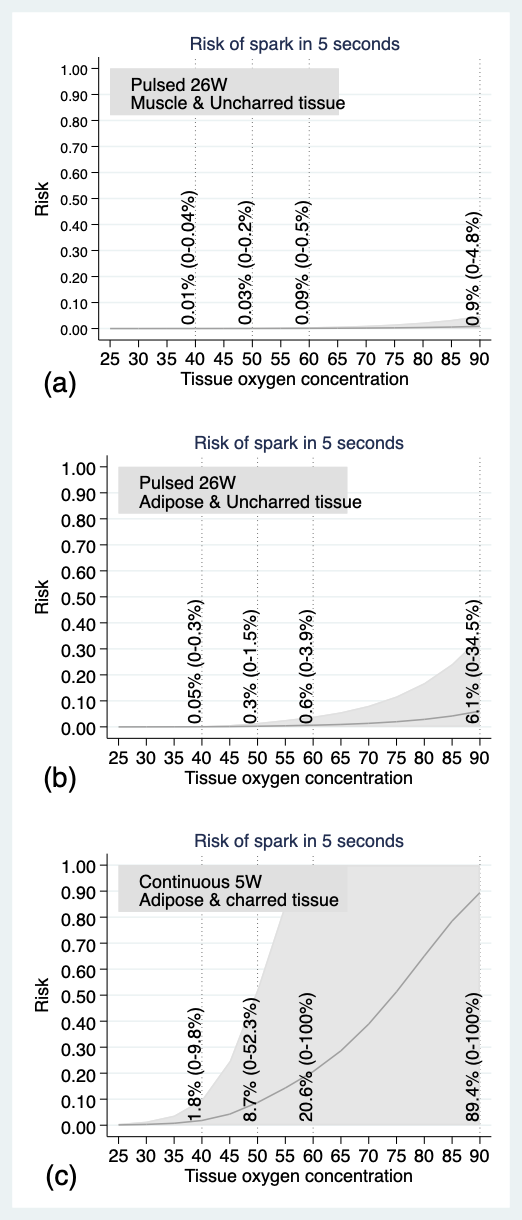

Supplement: Supplementary file 1 — Supplementary Figure S1. [file 41598_2021_4636_MOESM1_ESM.png]
