# Supplementary material for: Risk of airway fire with the use of KTP laser and high flow humidified oxygen delivery in a laryngeal surgery model
Source: Sci Rep. 2022 Jan 11;12:543. doi: 10.1038/s41598-021-04636-3 (PMC8752812; doi:10.1038/s41598-021-04636-3)
Supplement: Supplementary file 2 — Supplementary Legends. [file 41598_2021_4636_MOESM2_ESM.docx]

## Supplementary File

Supplementary Video 1: Lasing at FiO2 0.8, laser setting 2.6W continuous, fresh, adipose tissue. Flame at 34 seconds.

Supplementary Video 2: Lasing at FiO2 0.9, laser setting 5.0W continuous, charred, adipose tissue. Flame at 0 seconds.

Supplementary Figure S1. Restricted cubic spline of risk of spark at 5 seconds by combination of oxygen concentration, laser mode and tissue type based on experimental data. Percentages on the figures show the risks for oxygen concentration 40, 50 and 60 respectively. (a) Laser setting: 26W pulsed, tissue: uncharred muscle; (b) Laser setting: 26W pulsed, tissue: uncharred adipose tissue; (c) Laser setting: 5W continuous, tissue: charred adipose tissue.
